# Autogenous Costal Cartilage Grafting With Titanium Mesh Fixation for Comminuted Bilateral Zygomatic Arch Fractures: An Autobiographical Case Report

**DOI:** 10.7759/cureus.104857

**Published:** 2026-03-08

**Authors:** Richard M Cavero, Ines A Camejo, Adisley Triay, Dileisy Jimenez

**Affiliations:** 1 General Practice, Metro Dental Associates, Morristown, USA; 2 Dentistry, Private Practice, New York, USA; 3 Dentistry, Private Practice, Houston, USA

**Keywords:** autogenous cartilage, bilateral zygomatic, cartilage graft, comminuted fracture, facial trauma, fracture costal, midface reconstruction, titanium mesh fixation, zygomatic arch' maxillofacial surgery

## Abstract

Bilateral zygomatic arch fractures are uncommon but may result in significant midfacial deformity and functional impairment, particularly when fractures are comminuted and structural continuity is compromised. This report describes the surgical management of a 49-year-old male who sustained severe facial trauma following a mountain-climbing accident, resulting in comminuted bilateral zygomatic arch fractures and marked midfacial disfigurement. Due to the complexity of the injury and loss of structural support, autogenous costal cartilage grafting combined with titanium mesh fixation was selected for reconstruction.

Under general anesthesia, bilateral preauricular and infraorbital approaches were used to expose the fracture sites. Autogenous costal cartilage was harvested from the left costal cartilage (typically sixth-eighth rib), contoured according to the anatomical requirements of both zygomatic arches, and positioned to restore midfacial contour. Titanium mesh and screws were applied to achieve rigid fixation and maintain structural stability.

Short-term follow-up of three months demonstrated satisfactory healing without complications. Facial symmetry and midfacial projection were successfully restored, and the patient reported high satisfaction with the surgical results. The patient described in this report is also one of the authors, and this dual role is disclosed for ethical transparency. This case highlights autogenous costal cartilage grafting with titanium mesh fixation as a viable reconstructive option in complex bilateral zygomatic arch fractures when conventional reduction and fixation may be insufficient.

## Introduction

Zygomatic arch fractures represent a significant component of midfacial trauma and may lead to functional and esthetic complications if not properly treated. Zygomatic arch fractures represent a significant portion of midfacial trauma and may result from high-impact injuries such as motor vehicle accidents or falls [1]. The zygomatic arch plays a critical role in maintaining facial symmetry, mastication, and structural support of the midface [2]. High-impact facial injuries may result in complex or comminuted fractures that present reconstructive challenges for maxillofacial surgeons [3].

Conventional management of zygomatic arch fractures typically involves open reduction and internal fixation using plates and screws [4]. However, in cases of severely comminuted fractures with disruption of structural continuity, standard fixation techniques may be insufficient to restore facial contour and skeletal stability [5]. In such situations, reconstructive approaches utilizing autogenous grafts may provide improved structural reinforcement and esthetic outcomes [6].

Autogenous costal cartilage grafting has been widely employed in craniofacial and reconstructive surgery because of its biocompatibility, availability, and capacity to provide structural support [6]. When combined with rigid fixation methods such as titanium mesh, cartilage grafts may offer an effective option for restoring midfacial contour and stability in complex fractures [7]. This report describes a case of comminuted bilateral zygomatic arch fractures managed with autogenous costal cartilage grafting and titanium mesh fixation.

## Case presentation

**Figure 1:**
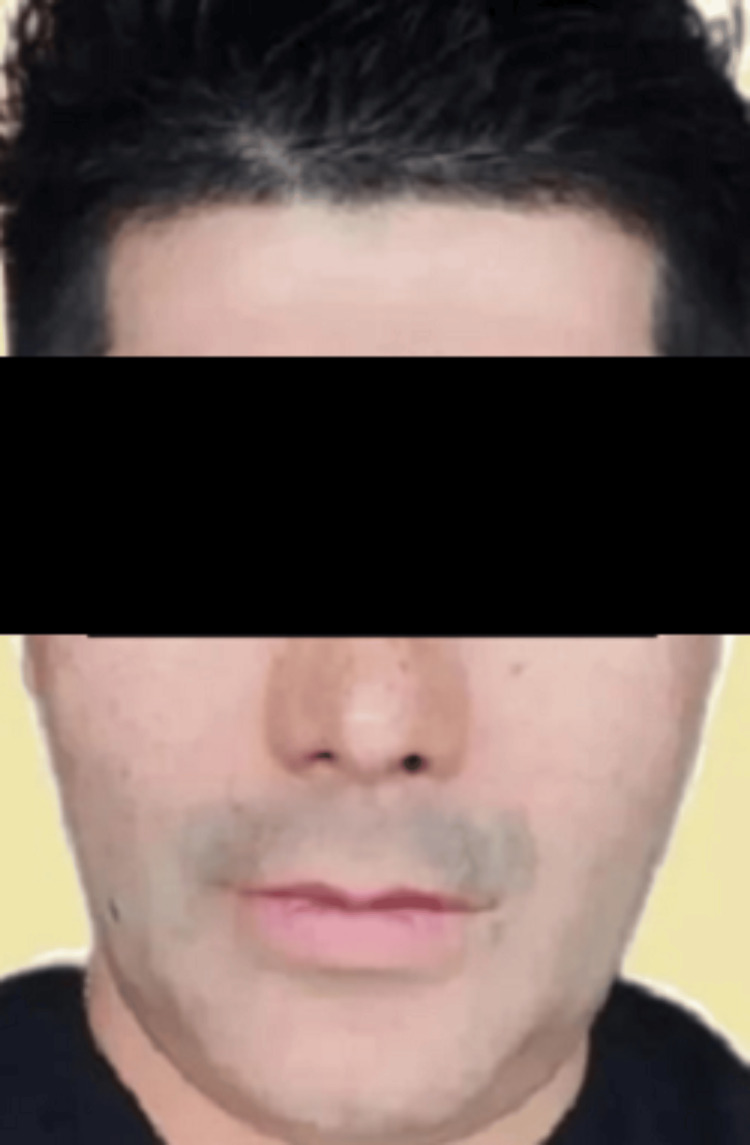
Pre-trauma facial appearance (baseline).

**Figure 2:**
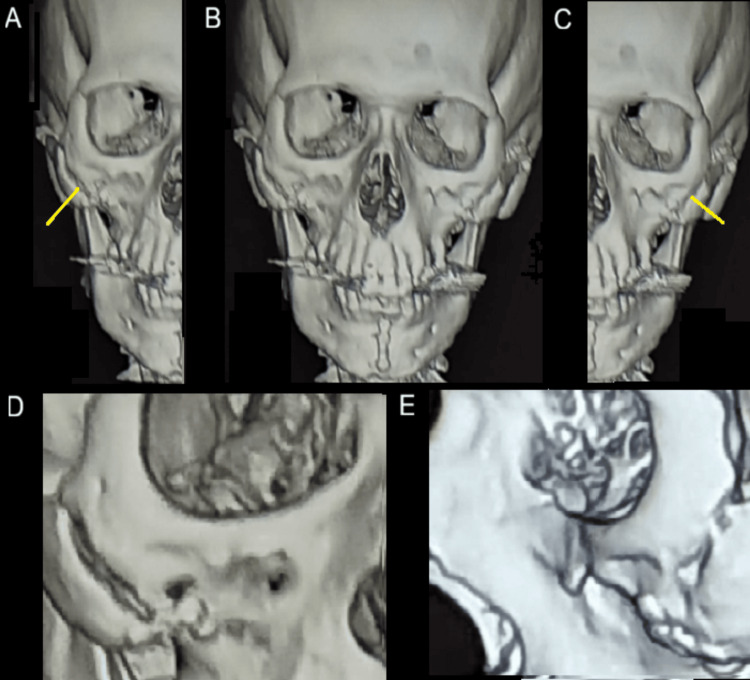
Preoperative radiographic images demonstrating bilateral comminuted zygomatic arch fractures. (A) Right zygomatic arch fracture. (B) Frontal view demonstrating bilateral zygomatic arch fractures. (C) Left zygomatic arch fracture. (D) Close-up view of the right zygomatic arch fracture. (E) Close-up lateral view of the right zygomatic arch fracture.

**Figure 3:**
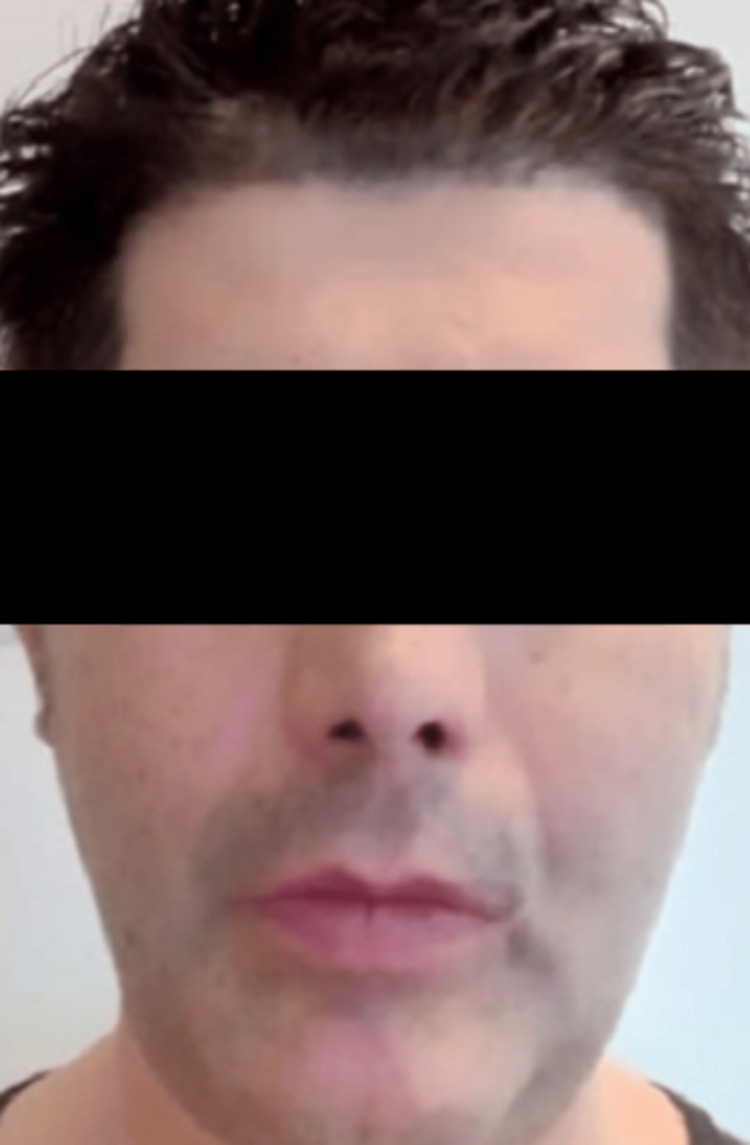
Post-trauma clinical deformity with bilateral swelling.

A 49-year-old male presented with severe facial trauma following a mountain-climbing accident in which he struck his face against rocks. A baseline pre-trauma facial appearance is shown in Figure 1. Radiographic evaluation confirmed comminuted fractures of both zygomatic arches with disruption of structural continuity (Figure 2). Post-trauma clinical examination revealed facial asymmetry and depression over the bilateral zygomatic regions consistent with bilateral zygomatic arch fractures (Figure 3).

The case was managed by an experienced maxillofacial surgery team with the objective of restoring both functional and esthetic facial anatomy. After comprehensive clinical and radiographic assessment and multidisciplinary planning, autogenous costal cartilage grafting was selected due to the comminuted nature of the fractures and insufficient native bone support for conventional fixation.

**Figure 4:**
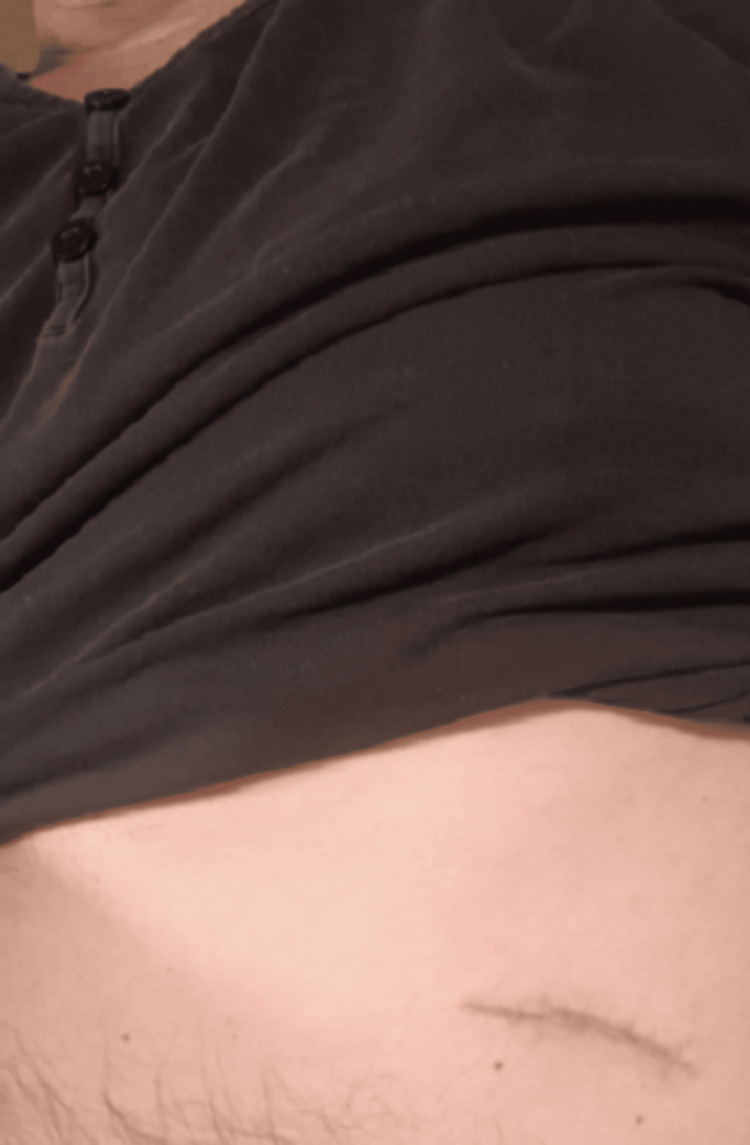
Harvesting of autogenous costal cartilage graft.

Under general anesthesia, surgical access was obtained through approximately 2 cm bilateral preauricular vertical incisions and an additional 2 cm suborbital incision placed along natural anatomical landmarks. A separate 4 cm incision was made in the left thoracic region for harvesting autogenous costal cartilage (Figure 4). The harvested cartilage was sculpted to match the anatomical dimensions required for bilateral zygomatic arch reconstruction.

**Figure 5:**
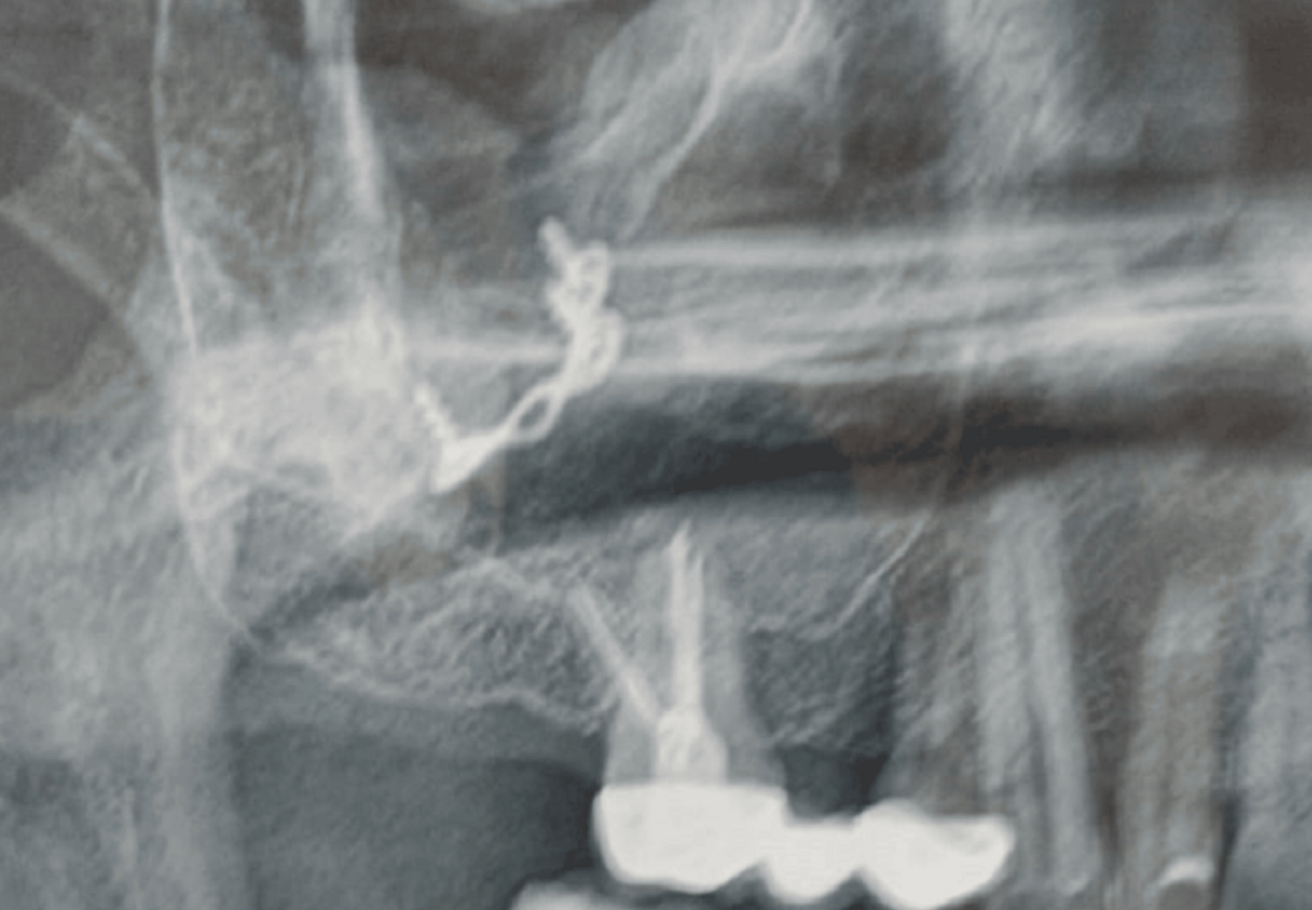
Intraoperative placement of autogenous costal cartilage graft secured with mini titanium plates and screws.

**Figure 6:**
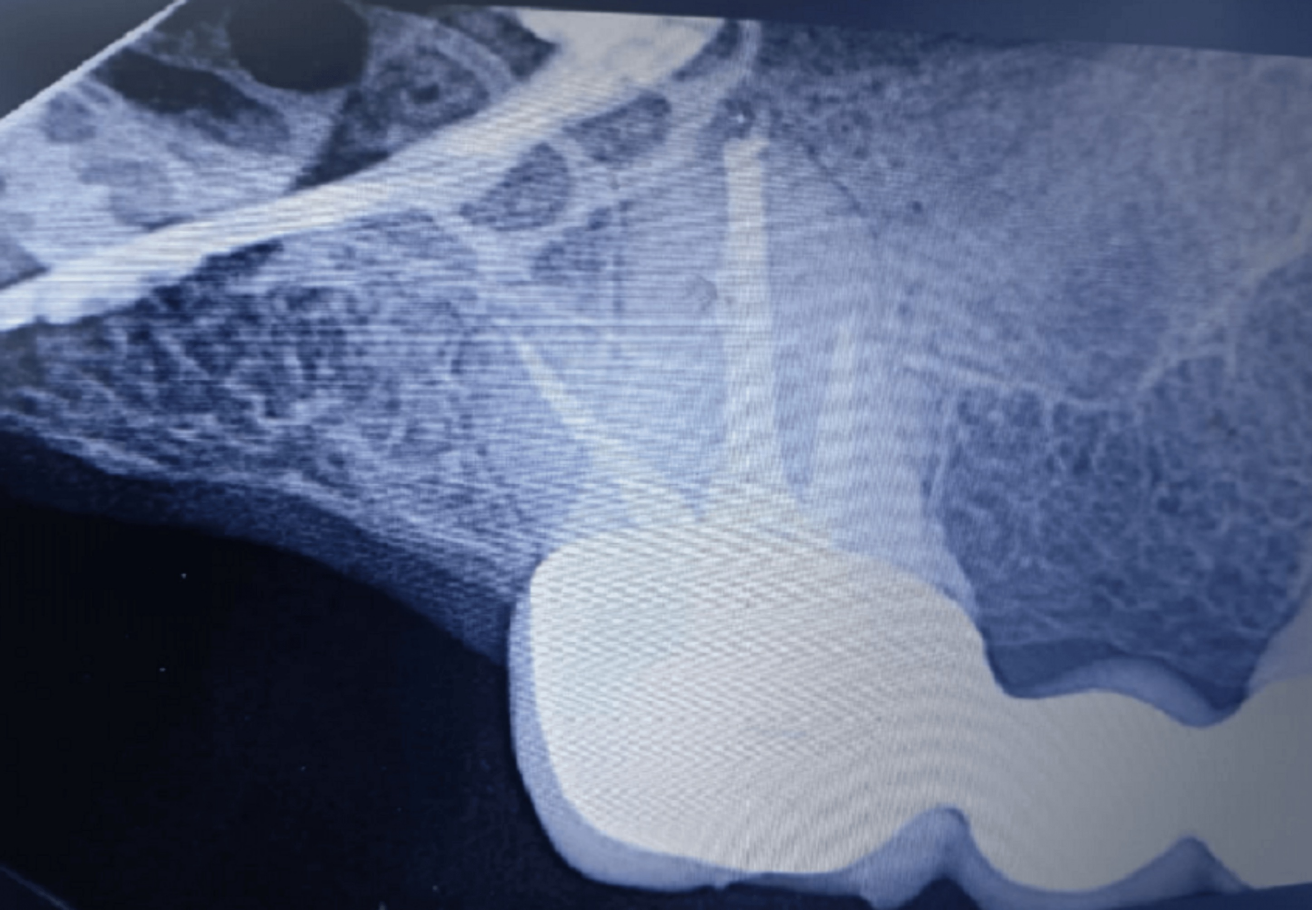
Reinforcement of the cartilage graft with titanium mesh and screw fixation to restore zygomatic contour.

The costal cartilage grafts were positioned to restore the contour and structural continuity of the zygomatic arches (Figure 5). Titanium mesh and screws were used to reinforce the grafts and provide rigid fixation, ensuring structural stability and appropriate anatomical alignment (Figure 6). This approach facilitated restoration of facial symmetry and midfacial support.

**Figure 7:**
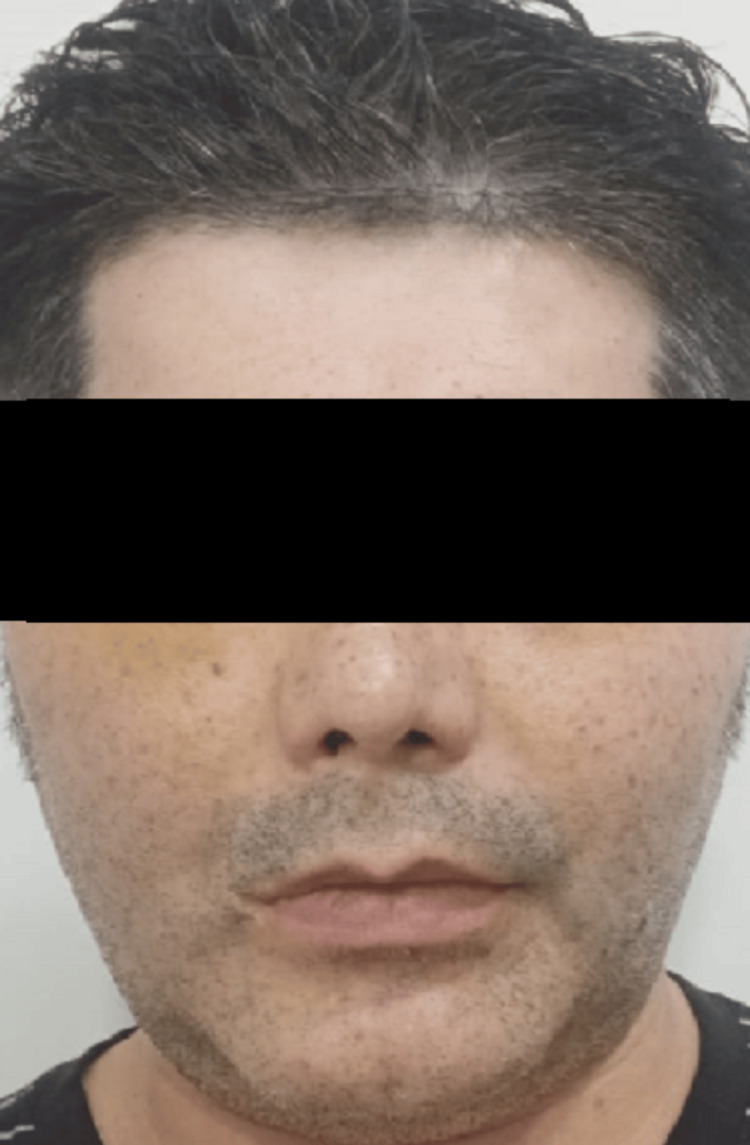
Early postoperative clinical appearance demonstrating appropriate wound healing. Frontal view demonstrating improvement in facial contour following bilateral zygomatic arch reconstruction. Mild residual facial asymmetry and soft tissue edema are present, consistent with the early postoperative healing phase.

**Figure 8:**
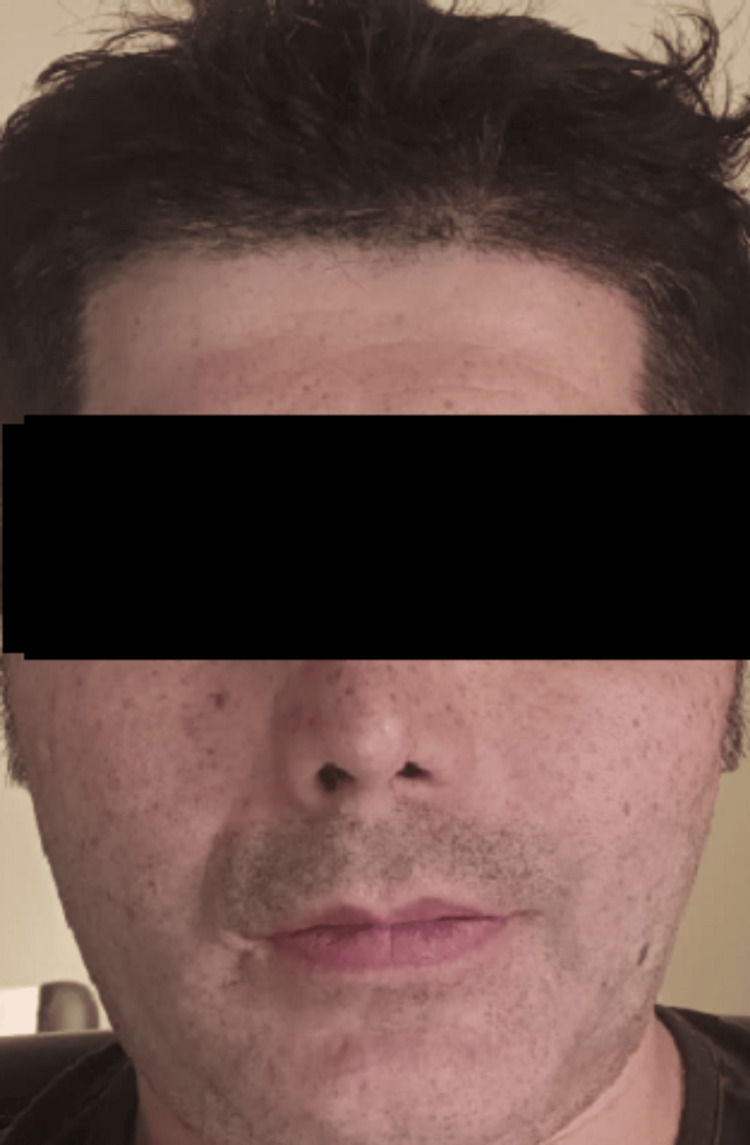
Postoperative clinical appearance after resolution of edema at three-month follow-up. Frontal view demonstrating improved facial symmetry and restoration of zygomatic contour following bilateral reconstruction.

Early postoperative clinical appearance demonstrated appropriate wound healing (Figure 7). Postoperative follow-up over three months demonstrated satisfactory healing without complications. Clinical assessment showed restoration of facial contour and symmetry (Figure 8).

Preoperatively, the patient received amoxicillin 500 mg, cephalexin 500 mg, and ibuprofen 400 mg administered orally every eight hours (three times daily) prior to the surgical procedure.

Following surgery, the patient was prescribed amoxicillin 500 mg, cephalexin 500 mg, and clindamycin 300 mg administered orally every eight hours (three times daily). In addition, omeprazole was prescribed once daily for gastric protection, dexamethasone 4 mg once daily for anti-inflammatory management, and ibuprofen 400 mg four times daily for pain control. The postoperative medications were continued for 14 days.

At follow-up, the patient reported restoration of facial symmetry and no current discomfort associated with the titanium mesh following the procedure.

Written informed consent was obtained from the patient for publication of this case report and accompanying clinical information. The patient described in this report is also one of the authors; this dual role is disclosed for ethical transparency. All clinical details were independently reviewed by the co-authors prior to submission.

## Discussion

Bilateral zygomatic arch fractures are less common than unilateral injuries but may result in significant functional and esthetic impairment when structural continuity is disrupted [8]. The zygomatic arch contributes to midfacial width and malar projection and serves as an attachment site for the masseter muscle; disruption may therefore lead to facial asymmetry and impaired mastication [1,8].

Standard management of zygomatic arch fractures typically involves closed reduction techniques for simple fractures or open reduction with internal fixation for displaced injuries [1,2]. Closed reduction methods such as Gillies elevation have demonstrated favorable outcomes in simple fractures; however, these approaches may be insufficient in severely comminuted injuries [3]. In cases of extensive fragmentation and loss of structural continuity, achieving stable fixation and precise contour restoration may be challenging [7].

Autogenous grafting materials have long been utilized in craniofacial reconstruction due to their biocompatibility and structural support properties [6]. Costal cartilage has been employed in midfacial augmentation and reconstruction because of its availability and adaptability to complex anatomical shapes [5,6]. In the present case, autogenous costal cartilage was selected to restore bilateral zygomatic arch continuity due to extensive comminution and the need for additional structural reinforcement.

Titanium mesh fixation has been reported to provide rigid stabilization in maxillofacial fractures while allowing contour adaptation [4]. The combination of autogenous cartilage and rigid fixation in this case provided structural stability and satisfactory restoration of midfacial contour without postoperative complications during follow-up.

This case supports previous findings suggesting that grafting combined with rigid fixation may represent a viable reconstructive alternative in complex zygomatic arch fractures when conventional fixation alone may be insufficient [4,7]. Careful preoperative planning, precise graft shaping, and stable fixation remain essential to achieving predictable functional and esthetic outcomes in challenging bilateral midfacial injuries.

## Conclusions

Comminuted bilateral zygomatic arch fractures present substantial reconstructive challenges due to disruption of structural continuity and alteration of midfacial contour. When conventional reduction and internal fixation techniques are inadequate, autogenous costal cartilage grafting combined with titanium mesh fixation may provide a stable and biocompatible reconstructive alternative. In the present case, meticulous preoperative planning and rigid stabilization resulted in satisfactory short-term functional recovery and restoration of facial symmetry without postoperative complications. However, as this report describes a single clinical case with limited follow-up, these findings should be interpreted cautiously. This approach may represent a viable option in selected cases of complex midfacial trauma; nevertheless, further studies are required to evaluate long-term outcomes and broader clinical applicability.
